# International Urogynecology consultation chapter 2 committee 3: the clinical evaluation of pelvic organ prolapse including investigations into associated morbidity/pelvic floor dysfunction

**DOI:** 10.1007/s00192-023-05629-8

**Published:** 2023-09-22

**Authors:** Heather Barbier, Cassandra L. Carberry, Päivi K. Karjalainen, Charlotte K. Mahoney, Valentín Manríquez Galán, Anna Rosamilia, Esther Ruess, David Shaker, Karishma Thariani

**Affiliations:** 1 Uniformed Services University of the Health Sciences, Bethesda, MD USA; 2 Department of Obstetrics and Gynecology, Warren Alpert Medical School of Brown University/Women & Infants Hospital, Providence, RI USA; 3 Department of Obstetrics and Gynecology, Hospital Nova of Central Finland, Jyväskylä, Finland; 4 Department of Obstetrics and Gynecology, Tampere University Hospital, Tampere, Finland; 5 Institute of Clinical Medicine, University of Eastern Finland, Kuopio, Finland; 6 The Warrell Unit, St Mary’s Hospital, Manchester, UK; 7 Female Pelvic Floor Unit, Clinical Hospital of Universidad de Chile, Santiago de Chile, Chile; 8 Urogynaecologist and Reconstructive Pelvic Floor Surgeon, Cabrini Hospital, Malvern, Victoria Australia; 9 Monash Health, Monash University Department of O&G, Hudson Institute of Medical Research, Melbourne, Australia; 10 Department of Obstetrics and Gynecology, University Hospital of Basel, Basel, Switzerland; 11 Rural Clinical School Rockhampton Australia, Mater Private Hospital Rockhampton Australia, University of Queensland, St Lucia, Australia; 12 Fellowship in Urogynaecology & Pelvic Reconstructive Surgery, Consultant Urogynaecologist, Centre for Urogynaecology & Pelvic Health, New Delhi, India

**Keywords:** Pelvic organ prolapse, Clinical evaluation, Urinary tract dysfunction, Gastrointestinal dysfunction

## Abstract

**Introduction and hypothesis:**

This manuscript from Chapter 2 of the International Urogynecology Consultation (IUC) on Pelvic Organ Prolapse (POP) reviews the literature involving the clinical evaluation of a patient with POP and associated bladder and bowel dysfunction.

**Methods:**

An international group of 11 clinicians performed a search of the literature using pre-specified search MESH terms in PubMed and Embase databases (January 2000 to August 2020). Publications were eliminated if not relevant to the clinical evaluation of patients or did not include clear definitions of POP. The titles and abstracts were reviewed using the Covidence database to determine whether they met the inclusion criteria. The manuscripts were reviewed for suitability using the Specialist Unit for Review Evidence checklists. The data from full-text manuscripts were extracted and then reviewed.

**Results:**

The search strategy found 11,242 abstracts, of which 220 articles were used to inform this narrative review. The main themes of this manuscript were the clinical examination, and the evaluation of comorbid conditions including the urinary tract (LUTS), gastrointestinal tract (GIT), pain, and sexual function. The physical examination of patients with pelvic organ prolapse (POP) should include a reproducible method of describing and quantifying the degree of POP and only the Pelvic Organ Quantification (POP-Q) system or the Simplified Pelvic Organ Prolapse Quantification (S-POP) system have enough reproducibility to be recommended. POP examination should be done with an empty bladder and patients can be supine but should be upright if the prolapse cannot be reproduced. No other parameters of the examination aid in describing and quantifying POP. Post-void residual urine volume >100 ml is commonly used to assess for voiding difficulty. Prolapse reduction can be used to predict the possibility of postoperative persistence of voiding difficulty. There is no benefit of urodynamic testing for assessment of detrusor overactivity as it does not change the management. In women with POP and stress urinary incontinence (SUI), the cough stress test should be performed with a bladder volume of at least 200 ml and with the prolapse reduced either with a speculum or by a pessary. The urodynamic assessment only changes management when SUI and voiding dysfunction co-exist. Demonstration of preoperative occult SUI has a positive predictive value for de novo SUI of 40% but most useful is its absence, which has a negative predictive value of 91%. The routine addition of radiographic or physiological testing of the GIT currently has no additional value for a physical examination. In subjects with GIT symptoms further radiological but not physiological testing appears to aid in diagnosing enteroceles, sigmoidoceles, and intussusception, but there are no data on how this affects outcomes. There were no articles in the search on the evaluation of the co-morbid conditions of pain or sexual dysfunction in women with POP.

**Conclusions:**

The clinical pelvic examination remains the central tool for evaluation of POP and a system such as the POP-Q or S-POP should be used to describe and quantify. The value of investigation for urinary tract dysfunction was discussed and findings presented. The routine addition of GI radiographic or physiological testing is currently not recommended. There are no data on the role of the routine assessment of pain or sexual function, and this area needs more study. Imaging studies alone cannot replace clinical examination for the assessment of POP.

## Introduction

This report is part of a series of articles that are the product of the International Urogynecology Consultation (IUC), which is sponsored by the International Urogynecological Association (IUGA). This is a 4-year, four-chapter project, with 16 reports dedicated to reviewing and summarizing the world’s literature on pelvic organ prolapse (POP).

This report is from the 2nd year and chapter of the project, which is dedicated to the evaluation of POP. This year/chapter is divided into three reviews, the other two involve the radiographic evaluation of POP and the use of patient-reported outcomes (POP condition-specific quality-of-life questionnaires) in the evaluation of POP. This report focuses on the clinical evaluation of women with POP and describe how to use the physical examination to describe pelvic organ support or prolapse. In addition, the associated testing to evaluate comorbid conditions of the urinary and gastrointestinal tracts (GITs) is described and evaluated. Radiographic testing to evaluate comorbid lower urinary tract and gastrointestinal conditions is part of this report.

It is recommended that every patient with POP has a thorough clinical examination. Describing and evaluating the patient for POP, although it at first seems straightforward, is in fact very complex. First, there are several classification systems currently in use to describe and quantify POP. The clinician is then left to determine the relative benefits of using one system over another. In addition, it is recognized that many patients with POP often have pelvic floor comorbidities involving other pelvic/abdominal organ systems [1]. Choosing how best to use clinical resources to properly investigate these conditions in patients with POP can be confusing. In addition, the interpretation of test results in a patient with POP may be different than interpretation of the same studies in a patient with normal pelvic organ support. Finally, this paper addresses the question as to which additional testing is necessary and should be routine versus which testing should only be performed if there are associated symptoms present. This review is not meant to be an exhaustive paper regarding the evaluation of lower urinary tract or gastrointestinal symptoms in women, except as they are uniquely influenced by POP.

In this review, the components of a clinical examination and the conditions under which they should be performed are assessed and the best practices described. Any additional testing of co-morbid conditions that should be routinely undertaken, and the conditions under which they are best performed, are evaluated and the best practices described. Knowledge gaps and areas that require further study are also noted.

## Materials and methods

**Fig. 1:**
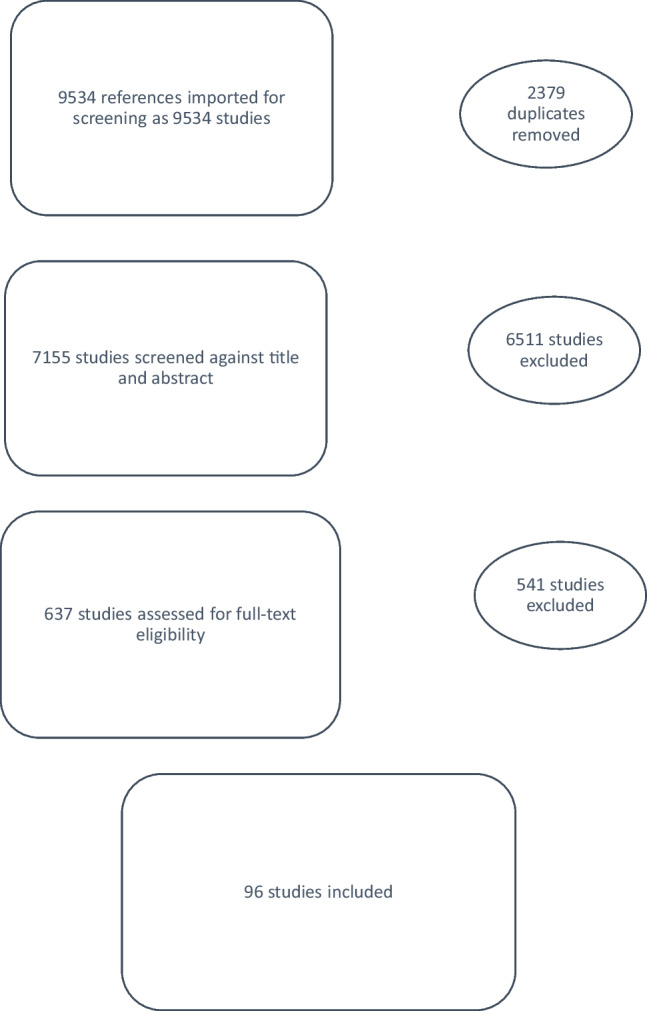
Preferred Reporting Items for Systematic Reviews and Meta-Analyses diagram for prolapse and examination findings

This manuscript is a narrative review that includes a systematic search of the literature using terms from the PubMed and Embase databases (January 2000 to August 2020). Only human studies involving adult women and limited to the English language were included. The terms for searching the literature were developed by the authors of this report and were presented to the IUGA membership at the annual scientific meeting in 2020; progress was reported at subsequent meetings. These are shown in Table 1 the titles and abstracts were reviewed using the Covidence database to determine whether they met the inclusion criteria. In the event of uncertainty, this was discussed at regularly scheduled meetings. The manuscripts were next reviewed for suitability using the Specialist Unit for Review Evidence checklists for cohort, cross-sectional, and case–control epidemiological studies. This was done to assess data presentation, population description, and bias. Only studies that included populations with clear definitions of patients with symptomatic POP, which described examination findings, were included. The full-text manuscripts were extracted and then reviewed. Those manuscripts that qualified were reviewed in depth and the process is summarized in the Results section (Fig. 1).

**Table 1:** Keywords used for searching the literature

Number	Evaluation of POP	Evaluation of LUTS	Evaluation of GIT	Evaluation of pelvic floor muscle function, sexual function, and pelvic pain
1.	Genital prolapse	Assessment of urinary symptoms	Assessment of defecation symptoms	Assessment of sexual dysfunction
2.	Uterovaginal prolapse	Urinalysis	Proctoscopy	Vaginal laxity
3.	Cystocele	Urinary incontinence, stress/cough stress test	Digital anorectal examination	Pelvic floor muscle strength
4.	Cystourethrocele	Post-void residual	Anal sphincter tone	Oxford Scale
5.	Anterior wall prolapse	Uroflow	Digital rectal examination	Clitoral sensation
6.	Rectocele	Urodynamics or urodynamic studies	Bowel diary	Blood flow
7.	Posterior wall prolapse	Cystometry	Bristol Stool Chart	Assessment of pelvic pain
8.	Enterocele	Pressure-flow study	Sigmoidoscopy	Evaluation of pelvic pain
9.	Recto-enterocele	Occult stress incontinence	Anorectal manometry	Cotton-swab test
10.	Perineocele	Bladder diary	Defecography	Sensory examination
11.	Procidentia	Frequency volume chart	Defecography with MRI	Trigger points
12.	Apical prolapse	Pad-weight test	Rectal prolapse	Pelvic floor muscle tenderness
13.	Vault prolapse	Cystoscopy	Intussusception	Pelvic floor resting tone
14.	Cervical elongation	Urethral mobility		Neuromuscular examination
15.	Pelvic organ prolapse	Q-tip		
16.	Uterine prolapse	Cotton swab test		
17.	Anterior compartment prolapse	Pessary reduction test		
18.	Posterior compartment prolapse	Urethral pressure profilometry		
19.	Perineal descent	Leak point pressure		
20.	Joint hypermobility and prolapse	Detrusor overactivity		
21.	Striae	Non-obstructive voiding difficulty		
22.	Urethral mucosal prolapse			
23.	Paravaginal defect			

*POP* pelvic organ prolapse, *LUTS* lower urinary tract symptoms, *GIT* gastrointestinal tract

## Results

The search strategy found 11,242 abstracts, which were reviewed and led to the extraction of 940 full-text articles, of which 220 articles were used to inform this narrative review. The results and the PRISMA figure for each are reported in three areas:

1. Clinical physical examination 
2. The urinary tract (LUTS), and
3. The gastrointestinal tract (GIT).

Other comorbid conditions such as pain and sexual dysfunction are better evaluated and recorded using patient-reported outcomes, which are covered in a separate manuscript of the IUC [2].

### Clinical physical examination of a woman with POP

**Fig. 2:**
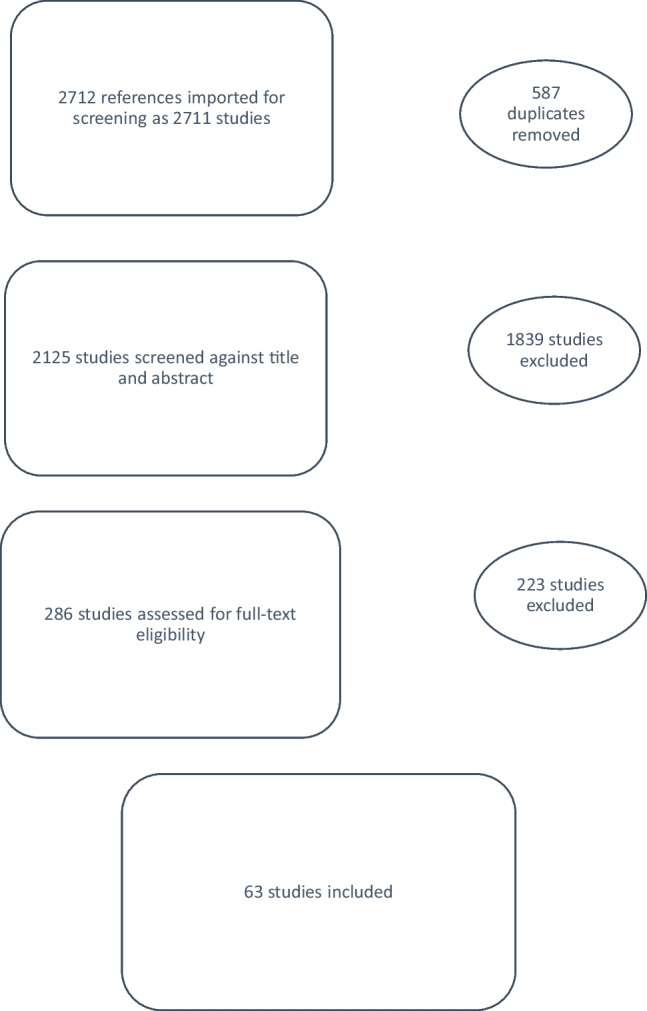
Preferred Reporting Items for Systematic Reviews and Meta-Analyses diagram for lower urinary tract symptoms

A review of the existing literature on the examination of a patient with POP and the impact of various parameters on the examination findings was performed. The initial search identified more than 7,155 abstracts of which around 96 studies were included in the final review (Fig. 2) This review of the clinical examination is divided into four sections:

1. General aspects of examination of a woman with POP
2. Examination of the anterior compartment
3. Examination of the posterior compartment
4. Examination of the apical compartment

#### General aspects of examination of a woman with POP

##### Methods to describe/quantify examination of POP

A variety of systems have been devised to classify and quantify POP. Eight studies focused on assessing the reliability and reproducibility of various staging systems (Table 2). It was found that the Baden–Walker Halfway Grading System had moderate reproducibility, making it unsuitable for clinical care or research [3]. The Pelvic Organ Prolapse Quantification (POP-Q) system, on the other hand, was found to have good interobserver agreement and was found to be particularly useful in the research setting [4].

**Table 2:** Results of studies assessing the different staging systems for pelvic organ prolapse

Staging system	Number of studies	Interobserver repeatability	Intersystem agreement with POP-Q	Validity	Simplicity/complexity
Baden–Walker	2	Moderate (kappa 0.50)	Fair to moderate	+	–
POP-Q	1	Good	–	+	Complex
Simplified POP-Q	3	Perfect (kappa 0.87)	Good	+	Simple
Eye ball POP-Q	1	Perfect (kappa 0.84)	Good	+	Simple for physicians well versed in standard POP-Q

*POP-Q* Pelvic Organ Prolapse Quantification

Owing to the complexity of the POP-Q, a simplified POP-Q (S-POP) system was devised. This system retains the ordinal stages of the POP-Q but simplifies the terminology and reduces the number of points measured. Three studies evaluated the validity, interobserver agreement, and inter-system agreement between the simplified POP-Q and POP-Q [5–7]. The authors concluded that a substantial intersystem association exists between S-POP and POP-Q, and S-POP, being simpler, may be more applicable to clinical practice worldwide. It was also found that the simplified POP-Q system retains its inter-examiner agreement across centers of varying degrees of expertise and is a valid, user-friendly alternative to POP-Q. For a complete description of the POP-Q please refer to the article by Bump et al. [8]. For a complete description of the S-POP please refer to the article by Swift et al. [9].

One study described and evaluated the validity of the novel “eye-ball” POP-Q technique (POP-Q by estimation) [10]. In this technique, the points along the anterior and posterior vaginal walls (Aa, Ba, Ap, and Bp) and on the perineum genital hiatus (GH) and perineal body (Pb) were visually estimated. Determination of vaginal depth (total vaginal length, or TVL) and apical descent (points C and D) were assessed by both visual estimation and palpation with the examiner’s dominant hand. The authors suggested that estimating POP-Q values provided comparable results to measuring them when performed by physicians well versed with the standard POP-Q.

##### Impact of various parameters on POP examination

When examining a patient with suspected POP, it is critical that the examiner sees and describes the maximal extent of the POP as experienced by the woman. This may be impacted by many variables including the patient’s age, parity, body mass index (BMI), position, bladder volume, rectal fullness, the timing of the day of the examination, examination performed at rest or Valsalva/straining, and effect of anesthesia in the case of examination in operating rooms. The correlation of examination findings with these variables was examined separately in nine studies. The conclusions of these are summarized below.

1. Age, parity, and BMI: there is no literature on how any of these impacts the ability of a woman to aid in her examination to identify the bothersome extent of her POP.

2. Bladder volume and rectal fullness: the effect of bladder volume on examination of POP was evaluated by two studies [11, 12]. Both concluded that the maximal extent of POP should always be assessed with an empty bladder. This could be because a full bladder does not allow maximal straining and also distorts the anatomy of the vaginal wall, especially of the anterior and central compartments. Similarly, a full rectum may cause confounding of findings by competing for space. One study commented that all patients with POP should be examined with an empty rectum if possible [13]. However, there is a lack of evidence to support this.
3. Patient position: there is a lack of standardized recommendations regarding patient position during a POP examination. Three studies examined the effect of patient position on the staging of POP [14–16]. It was found that the severity of POP demonstrated is greater when the examination is done in the upright position on a birthing chair or in the standing position rather than in the supine or lithotomy position. The inter-observer repeatability and correlation with the quality of life scores were also greater for examination findings in the upright position. In cases where the examination is not possible in an upright position, validation of POP-Q in a left lateral position was also assessed and the authors found a high degree of inter-observer reliability of POP-Q findings in this position [12].
4. Time of examination: the effect of the time of the day (morning versus afternoon) on POP-Q measurements, was assessed in a prospective observational study on 32 subjects [17]. No correlation was found between time of the day and extent of POP on examination. The authors concluded that for patients complaining of POP extending beyond the hymen there is no need to repeat an examination late in the day to confirm the full extent of prolapse.
5. Rest or straining: one study examined the predictive value of GH and Pb measurements obtained at rest and with straining for signs and symptoms of POP[18]. GH and Pb measured on straining were consistently stronger predictors of prolapse symptoms and objective prolapse (by clinician examination and by ultrasound) than at Gh and Pb measured at rest.
6. Anesthesia/neuromuscular blockade: the effect of neuromuscular blockade on POP staging was examined by one study [19]. It was found that neuromuscular blockade during anesthesia led to a significant increase in POP-Q measurements, especially in the apical compartment. The authors highlighted that in asymptomatic women with up to stage II POP, the surgical procedure should be limited to that planned preoperatively rather than allowing intraoperative findings to affect surgical management.
7. Role of cervical traction in prolapse examination: one study compared the degree of uterine prolapse between POP-Q with cervical traction and POP-Q in the standing position. They also assessed patient-reported pain and acceptability scores between the two examinations [20]. The median point C in the standing position was −4 (−7 to +2) and with cervical traction −0.5 (−3 to +4). Forty percent reported visual analog scale (VAS) pain scores of ≥5 under examination with cervical traction. Surprisingly, there was no significant difference in acceptability scores between the groups.

##### Relation of POP stage to GH length, Pb, and TVL

Two studies were aimed at describing the relationship between GH and Pb measurements with increasing POP stage [21, 22]. It was found that as the extent of POP increases, GH measurements also increased until stage 4 POP, where mean GH decreased. Also, the POP-Q measurement GH ≥ 3.75 cm is highly associated with and predictive of apical vaginal support loss. One study found that measurement of the TVL improved the correlation between the C-point measurement and POP symptoms [23].

##### Evaluation of pelvic floor muscle function in women with POP

Different methods have been used to study the pelvic floor muscle function (PFMF) and its correlation with severity of POP and pelvic symptoms. One study assessed whether POP severity, pelvic symptoms, quality of life, and sexual function differ based on PFMF (assessed by the Brink scale score; Table 3) by re-analyzing preoperative assessments of 317 of the 322 women enrolled in the Colpopexy and Urinary Reduction Efforts (CARE) trial [24]. They found that women with the highest Brink scores (*n*=75), suggesting enhanced pelvic floor muscle tone, had less advanced POP and smaller GH measurements, than those with the lowest Brink scores (*n*=56), suggesting weak pelvic floor muscle tone.

**Table 3:** Brink scoring system

Muscle function dimension	Score
Squeeze pressure	1 = None
	2 = Weak squeeze, felt as a flick at various points along the finger surface: not all the way around
	3 = Moderate squeeze; felt all the way around the finger surface
	4 = Strong squeeze; full circumference of fingers compressed
Muscle contraction duration	1 = None
	2 = Less than 1 s
	3 = Greater than 1 s but less than 3 s
	4 = Greater than 3 s
Vertical displacement	1 = None
	2 = Finger moves anteriorly
	3 = Whole length of finger move anteriorly
	4 = Whole fingers move anteriorly, are gripped, and pulled in
Total	Range 3–12

Two other studies tested the correlation between the PFMF, using the Oxford grading scale (Table 4), and the severity of POP. In one study 1,037 women were evaluated by assessing the POP-Q and the Oxford assessment of the PFMF. The muscle contraction was graded according to the modified Oxford grading system (Table 4): 0 = no contraction, 1 = flicker, 2 = weak, 3 = moderate, 4 = good, 5 = strong [25]. The levator hiatus (LH) size and GH were measured by digital examination [26]. Severity of POP correlated moderately with GH (r = 0.5, *p*<0.0001) and with LH (transverse r = 0.4, *p*<0.0001; longitudinal r = 0.5, *p*<0.0001), but weakly with the modified Oxford grading scale (r = 0.16, *p*<0.0001). In the second study, it was seen that POP stage had a significant influence on effective involuntary pelvic floor muscle contraction to counteract a sudden increase in intra-abdominal pressure during coughing. Women with POP stages II or more were significantly less able to achieve effective involuntary muscle contraction during coughing (which resulted in stabilization of the perineum; 37.7%) than women without POP (75.2%) [27].

**Table 4:** Modified Oxford Grading scale for pelvic floor muscle (*PFM*) strength

Grading	Description
0	No discernible PFM contraction
1	Very weak PFM contraction
2	Weak PFM contraction
3	Moderate PFM contraction
4	Good PFM contraction
5	Strong PFM contraction

##### Neurological examination in women with POP

There are very few data on neurological assessment in patients with POP. In a case–control study, the vaginal and clitoral sensory thresholds were assessed in 66 women with (*n*=22) and without POP (*n*=44) using a thermal and vibration Genito-Sensory Analyzer [28]. They found that women with POP exhibited significantly lower sensitivity in the genital area to vibratory and thermal stimuli than women without POP.

##### Association of spine curvature with POP and bony dimensions of the pelvis

Three studies evaluated the relationship of spinal curvature with POP. One study assessed the relationship of spinal curvature and POP, specifically, the loss of lumbar lordosis or pronounced thoracic kyphosis in 363 patients with symptomatic POP [29]. They found that patients with abnormal spinal curvature were 3.2 times more likely to develop POP than patients with a normal curvature (odds ratio, 3.18; 95% confidence interval, 1.46 to 6.93; *p*=0.002) and identified an abnormal change in spinal curvature as a significant risk factor in the development of POP. In the other two studies no differences in the mean T or L spine angles were found between women with and those without POP symptoms (*p*≥0.05) and bony dimensions on MRI at the level of the pelvic floor in matched cohorts were similar [30, 31].

#### Examination of different pelvic compartments in POP

##### Examination of anterior vaginal wall compartment for paravaginal defects

With respect to the clinical examination of the anterior vaginal wall defects, using the standardized POP-Q examination and a clinically defined technique for describing the presence of paravaginal defects, right and/or left lateral, central or superior defects have been described. To differentiate a midline or central defect from a paravaginal defect, an index finger or ring forceps must be placed vaginally toward each ischial spine separately. If the prolapse becomes reduced, the woman is clinically diagnosed with a paravaginal defect on that side. In a prospective study, the sensitivity to detect left, right, and bilateral paravaginal defects was reported to be 48%, 40%, and 23.5% respectively, whereas the specificities for each side were 71%, 67%, and 80% respectively compared with intraoperative findings. The overall prevalence of paravaginal defects in patients with at least POP-Q stage II POP of the anterior vaginal wall was 38% [32].

Another study assessed the inter-examiner and intra-examiner reliability of the evaluation of the anterior vaginal wall, including the evaluation of paravaginal defects, using the POP-Q examination and a standardized evaluation of paravaginal defects [33]. The clinical examination of anterior vaginal wall support defects displayed poor inter-examiner and intra-examiner agreement. Overall inter-examiner agreement was 42%, with a kappa of 0.16.

##### Correlation of anterior and apical compartment prolapse

The relationship or coexistence of anterior vaginal wall prolapse with apical prolapse was investigated in one study [34]. Women with a POP-Q Point Ba value ≥ −1 were retrospectively analyzed for the presence of apical POP defined as POP-Q point C value ≥ −3. The finding of POP-Q stage II or greater anterior vaginal wall prolapse was highly suggestive of clinically significant apical vaginal descent to −3 cm or greater. Furthermore, as the anterior vaginal wall POP-Q stage increased, the predictive value of apical POP increased. In women with POP-Q stage II anterior vaginal wall prolapse there was associated apical descent (defined as POP-Q point C ≥ −3) in 42%; in stage III anterior vaginal wall POP, apical descent was found in 85%; and in POP-Q stage IV anterior vaginal wall POP it was 100%.

#### Examination of the posterior compartment and the need for a rectovaginal examination

Three studies were identified that specifically evaluated the posterior vaginal wall and its relationship to GI dysfunction. A prospective cohort study used a variety of validated questionnaires and standardized examination measures, including Bp, AP, GH, and Pb, transverse GH, Pb at rest, with strain in addition to a “pocket” noted on rectal examination [35]. Inter- and intra-rater reliability for these were assessed by two independent examiners. This study demonstrated the reliability of these measurements of the posterior vaginal compartment and a weak association between obstructed defecation and pelvic organ prolapse.

Another study evaluated the association between defecatory symptoms such as constipation, painful defecation, fecal incontinence, and flatus incontinence and posterior vaginal wall examination using the POP-Q and by defecography [36]. The authors found no association between defecation disorders and posterior wall prolapse (evaluated by POP-Q) or rectocele (assessed by defecography) and that clinical examination missed most enteroceles. They concluded that most anatomical measures of posterior compartment prolapse are reliable and reproducible; however, they do not correlate well with defecatory symptoms.

One study assessed the evaluation of the rectovaginal septum (RVS) using a digital rectal examination [37]. The authors concluded that extending the clinical examination of prolapse to include rectal examination to palpate defects in the RVS may reduce the need for a defecatory proctogram or ultrasound for the assessment of obstructive defecation and may help to triage patients in the management of posterior compartment prolapse. Larger rectoceles were easier to identify and true rectoceles may be best diagnosed by rectal examination.

#### Examination of the apical compartment

##### Normal values for the apical component of the POP-Q

One study assessed normal values for the apical component of the POP-Q (points C, D, and TVL) in asymptomatic women by re-analyzing data from the original 2005 Pelvic Organ Support Study using a data set of 1,011 women [38]. In patients without POP defined as all POP-Q points above the hymenal remnants, they found mean POP-Q values to be: point C (vaginal cuff) −7.3 ± 1.5 cm, point C (cervix) −5.9 ± 1.5, point D −8.7 cm ± 1.5 cm, TVL (no hysterectomy) 9.8 cm ± 1.3 cm, and TVL (hysterectomy) 8.9 cm ± 1.5 cm.

##### Clinical evaluation of cervical elongation

A study evaluating 39 consecutive patients who had a preoperative POP-Q and a pathology report that documented the cervical length was performed. The comparison was between estimated cervical length (eCL) on the preoperative POP-Q (by subtracting point D from point C) to the actual cervical length (aCL) reported in the pathology report. The authors found a statistically significant difference between the eCL (mean 5.6 ± 2.91 cm) and the mean aCL (3.2 cm ± 0.99; *p*<0.0001). However, there was not a statistically significant difference between the eCL and aCL in patients whose prolapse was proximal to the hymen (3.5 ± 2.21 cm vs 3.1 ± 1.06 cm; *p* = 0.475). The authors concluded that the cervical length measured using POP-Q may not be accurate at more advanced stages of prolapse [39].

##### Apical descent in the office compared with evaluation in the operating room

One study compared the assessment of apical prolapse in the office and assessment in the operating room [40]. The office assessment was conducted using a standard POP-Q examination with measurement at straining. The intraoperative assessment was performed by placing traction on the cervix with a tenaculum. The mean difference in the C point between the two clinical settings was 3.5 cm with a difference of ≥5 cm in 33% of subjects. Of note, the mean difference was larger for women with lesser stages of prolapse: 5.8 cm at stage 1, 3.0 cm at stage 2, and 1.4cm at stages 3/4 (*p*<0.001). A difference of ≥5 cm in point C with cervical traction was more commonly noted with lower stages of prolapse; it was noted in 70.3% of women with stage 1 versus only 9.3% of women with stage 2, and 8.5% in women with stage 3 (*p*<0.001).

##### Association of posterior and anterior prolapse with apical support

Two studies evaluated the association of anterior and posterior compartment prolapse with apical support. In the first study the authors found that the mean point C location was −6.9 ± 1.5 (mean ± standard deviation) in control patients without POP. In patients with posterior prolapse point C was −4.7 ± 2.7 cm. In patients with anterior prolapse point C was −1.2 ± 4.1 cm, *p* values were <0.001 for all comparisons [41]. The authors concluded that posterior-predominant prolapse involved threefold less apical descent than in patients with anterior-predominant prolapse. In the second study the authors analyzed 196 subjects and performed a standard POP-Q examination, and then assessed anterior and posterior prolapse in each subject before and following support of the apex using the posterior half of a Graves speculum [42]. Their POP-Q stages before apical support were stage 2 prolapse in 36% of patients, stage 3 in 55%, and stage 4 in 10%. With simulated apical support from the Graves speculum, point Ba changed to stage 0 or 1 in 55% and Bp changed to stage 0 or 1 in 30% (*p*<0.001 for both). The mean change in Ba with apical support was 3.5 ± 2.6 cm and for point Bp the mean change was 1.9 ± 2.9 cm (*p*<0.001). These findings suggest a greater relationship between the anterior vaginal wall and apical prolapse.

##### Summary of clinical examination of a woman with POP

The clinical evaluation of a patient suspected of having POP by presenting symptoms should start with a thorough pelvic examination. The POP-Q system is the most studied POP classification system for describing and quantifying POP. It should be used clinically in settings where clinicians have extensive experience and comfort in its use. In clinicians with extensive experience, POP-Q values can often be reliably and adequately obtained by “eyeballing.” The POP-Q should be used in all research settings. In settings that do not have extensive experience with the POP-Q, or in settings that find it cumbersome to use, substituting the S-POP is acceptable as a means of describing and quantifying POP. The use of other systems currently in the literature should be discouraged unless more literature is published demonstrating their utility.

To optimally perform a physical examination on a patient with suspected POP several parameters should be met:

1. The subject should have an empty bladder (and empty rectum, if possible.
2. If the subject cannot confirm the extent of their POP by examination in the supine or left lateral position, the examination should be repeated in a more upright or standing position.
3. The time of day of the examination is not important in most cases.
4. The examination should be done during straining or coughing.
5. Cervical traction or examination under the effects of a neuromuscular blockade may overstate the degree of apical POP and should not be relied upon.

Other parameters of a thorough pelvic examination and imaging for pelvic anatomy are less well investigated but may provide some clinical assistance in planning therapy.

1. Noting the dimensions of the GH or vaginal introitus plays a role in the evaluation of a patient with POP. A large GH as documented by a POP-Q examination ≥ 3.75 cm is associated with greater degrees of POP. Understanding what information this provides to the clinician in staging and quantifying POP is less clear and requires more study. Of note, recording the size of the GH is part of the POP-Q but not the S-POP.
2. A greater pelvic floor muscle contraction strength has been associated with less severe POP by both POP-Q examination and various POP symptom scores. In addition, patients with POP appear to have some degree of neurological deficit in other pelvic structures. Therefore, evaluating and recording pelvic floor muscle contraction strength and the presence or absence of neurological deficits, although encouraged, does not currently play a recognized role in the evaluation or quantification of POP.
3. Evaluation of the spine in patients with POP may lead to better understanding of the epidemiology and pathophysiology of POP but does not play any specific role in the evaluation of patients with POP.
4. Clinical examination to identify and characterize site-specific defect of the anterior vaginal wall prolapse has not been studied enough to draw robust conclusions. However, although reporting these clinical findings may aid the individual surgeon in preoperative planning, is too nonspecific for widespread adoption into current clinical grading schemes.
5. Evaluation of posterior vaginal wall prolapse can be complemented by a rectovaginal examination as there is evidence that it can help to distinguish between true rectoceles and enteroceles. There is poor correlation between posterior vaginal prolapse by clinical examination and GI dysfunction.
6. Evaluation and grading of apical (cervical/vaginal vault) POP is complex and currently there is very little information from which to draw clinically relevant information. It appears that in normal subjects the cervix (POP-Q point C) is 4.5 to 7.5 cm above the hymenal remnants, the posterior vaginal fornix (POP-Q point D) is 7 to 10 cm above the hymenal remnants, and in hysterectomized patients the vaginal cuff (POP-Q point C in hysterectomized patients) is 6 to 8.5 cm above the hymen. The TVL in patients with a uterus is 8.5 to 11 cm and in hysterectomized patients it is 7.5 to 10.5 cm. The determination of a cut-off point beyond which apical values represent true POP or clinical symptomatic disease is unknown although any compartment prolapse at or beyond the hymen is more likely to be symptomatic.
7. Repeating a POP-Q examination under anesthesia often overestimates apical prolapse and although useful for surgical planning, currently should not be recommended. It is not known whether there is a long-term prognostic value for this apical assessment.
8. Using a tenaculum to provide traction on the cervix in the clinical setting can overestimate uterine prolapse, is deemed uncomfortable by patients, and therefore should be discouraged.

##### Further research

1. Future research needs to determine the predictive value of a large GH as a sign of impending POP that may require prophylactic therapeutic measures. Further, is a large GH a risk factor for POP or a side effect of having the vaginal bulge protruding through and physically dilating the vaginal opening?
2. Future research on what represents true uterine or vaginal vault prolapse is critical. There are some data on the normal range of values for POP-Q points C and D. However, what is not known is if patients with POP-Q point C and D values below these ranges but still above the hymenal remnants have a type of POP that requires therapeutic measures, particularly if that patient is undergoing surgery to correct anterior or posterior vaginal wall prolapse.
3. If a paravaginal defect is detected what is the role of anterior vaginal repair? To what degree does a paravaginal defect contribute to anterior vaginal wall recurrence?
4. Further study on how physical examination under the effects of neuromuscular blockade (anesthesia) affects future outcomes. For example, if a subject has significant cervical or apical POP identified in the operating room that was not noted during clinical physical examination, are they at a greater risk of future apical POP, particularly if nothing is done to address this apparent apical defect at the time of surgery for another form of POP?
5. Future research should better define the role of weak pelvic floor muscle tone or contraction strength as a predictor of the subsequent development of POP. A complete discussion of the role of pelvic floor muscle strength training and its role in treating POP will be included under another report in the IUC that has been published as part of this series entitled “International Urogynecology Consultation chapter 3 committee 2; conservative treatment of patients with pelvic organ prolapse: pelvic floor muscle training” [43].

### Assessment of lower urinary tract function in women with POP

A review of the existing literature on the assessment of lower urinary tract function in women with POP was performed. The initial search identified 2,711 titles and abstracts, of which 63 studies were included in the final review of this section (Fig. 2).

This section is presented in three sub-sections: the assessment of voiding dysfunction, assessment of detrusor overactivity (DO), and assessment of stress urinary incontinence (SUI).

#### Assessment of voiding dysfunction in women with POP

The prevalence of voiding dysfunction in women with prolapse varies depending on the definition but ranges from 6 to 60%. Assessment of voiding difficulty in women with prolapse was addressed in 11 papers. Six papers had voiding difficulty as the focus [44–49] , 4 papers addressed voiding difficulty as part of LUTS assessment [50–53], and 1 paper addressed the accuracy of ultrasound in measuring bladder volume [54]. Six themes were identified in these studies.

##### Post-void residual urine volume

Post-void residual urine volume (PVR) was the most utilized measure to define voiding dysfunction in the studies reviewed; however, there was no agreement on the cut-off value at which retention was diagnosed ranging from 50 to 200 ml, as shown in Table 5.

**Table 5:** Measures for the assessment of voiding difficulty

	Number of studies	Reference numbers of the studies
Post void residual volume	10	
>50 ml	1	(46)
>100 ml	6	(39, 41, 43–48, 47)
>150 ml	1	(38)
>200 ml	1	(42)
>25% of total bladder volume	1	(40)
Urin flow studies		
Q max	4	
<12 ml/s	1	(47)
<15 ml/s	3	(38, 42, 45)
Bladder outlet obstruction		
Pdet Max >40 cm H_2_O	1	(45)
Pdet Qmax >20 cm H_2_O with Qmax <12	1	(47)
Detrusor underactivity		
Bladder Contractility Index	1	(39)
Schafer’s grading	1	(50)
Pdet Max <10 cm H_2_O	1	(42)
Prolapse reduction during voiding assessment	3	(41, 42, 46)

*Qmax* maximum flow rate, *Pdet Max* maximum detrusor pressure as measured during pressure flow studies, *Pdet Qmax* pressure detrusor at maximum flow rate, *DU* detrusor underactivity

To find a cut-off value for PVR that could predict postoperative voiding trial results more accurately than a predetermined value of 100 ml, one study used a receiver operating curve, but no PVR value was better than 100 ml (the predetermined value used in the study) [49].

The accuracy of translabial ultrasound scan formulae used for PVR measurement in patients with prolapse was examined in one paper [54]. It found that the results obtained by the three published formulae correlated with the catheter-measured PVR.

##### Urine flow studies

These included free-flow studies (non-instrumented flow studies) and pressure-flow studies (instrumented urodynamic flow studies). Different measurements were used to define voiding dysfunction, as shown in Table 5.

One study [46] examined the correlation between free-flow and pressure-flow studies. It concluded that the peak and average flow rates in women with POP are dependent on voided volume and the correlation between free-flow and pressure-flow studies decreases as the prolapse stage increases.

##### Detrusor contractility measures

The concept of detrusor underactivity was addressed in two papers [45, 50] to predict the potential course of postoperative voiding difficulty. The Bladder Contractility Index (BCI), as defined by Abrams [55], was used in one paper [45]. BCI <100 was associated with higher PVR and a more severe stage of prolapse, but it failed to predict postoperative resolution of voiding difficulty. The second study [50] used a six-class grading of detrusor contractility based on Schafer’s nomograms [56]. They reported women with weak detrusor contractility having increased PVR in the immediate postoperative period, with resolution after 1 month.

##### Bladder trabeculation on cystoscopy

One study addressed the cystoscopic finding of trabeculation in women with POP. Trabeculations were scored from 0 to 4, representing increasing severity from none, slight, moderate, severe, and severe with diverticula. They reported significantly higher prevalence of symptoms of voiding difficulty and increased PVR (>100 ml) in women with any degree of trabeculations compared with women with no trabeculations [53].

##### Prolapse reduction in assessing voiding dysfunction

Prolapse reduction using a pessary or gauze pack was used to assess the impact of prolapse on voiding difficulty in three papers [47, 48, 52]. One study used pessary reduction of prolapse to predict postoperative resolution of voiding difficulties [47]. Authors reported that the resolution of voiding difficulty with pessary reduction of prolapse has 89% sensitivity and 80% specificity in predicting post-repair resolution [47]. In another study, pessary reduction of prolapse was used routinely in all patients while performing urodynamics [48] to assess voiding dysfunction and occult SUI. This resulted in the diagnosis of voiding dysfunction defined as post-void residual of >50 ml or 20% of voided volume in 27%, which reduced to 10% postoperatively. The authors did not test the value of pessary in predicting postoperative voiding dysfunction. The third study used vaginal packing for prolapse reduction and found that PVR decreased significantly after vaginal packing [52].

##### Risk factors for postoperative voiding dysfunction

Five studies looked at the assessment of potential risk factors to predict postoperative persistence of voiding dysfunction [45, 47–50]. In two studies, no potential risk factors were found [45, 50]. Three papers reported various potential risk factors to include history of diabetes, PVR >200 ml and detrusor pressure at maximum flow (Pdet Max) <10 cm H_2_O, all of which were found to have some impact on postoperative voiding dysfunction [48]. Persistence of voiding difficulty after pessary reduction of prolapse was associated with a 67% chance of persistent postoperative voiding difficulty [47]. Patient age was reported as the only risk factor for postoperative elevated PVR [49].

#### Assessment for detrusor overactivity in patients with POP

The effect of POP on detrusor overactivity (DO) was addressed in ten papers [50–53, 57–62]. Table 6 demonstrates the measures used to assess DO, the aim of assessment, and the use of prolapse reduction.

**Table 6:** Studies addressing detrusor overactivity (*DO*) in patients with pelvic organ prolapse (*POP*)

	Number of studies	Reference numbers of the studies
Method of assessing for DO		
Urodynamics (cystometry)	8	(44–46, 51–55)
Trabeculations on cystoscopy	1	(47)
Artificial neural network analysis	1	(56)
Aim of assessing for DO^a^		
Assessment for DO as co-morbidity with POP	3	(46, 47, 56)
Assessing the value of urodynamics in patients with POP	5	(44, 45, 52, 53, 55)
Assessment for risk factors predicting DO post-repair	3	(44, 51, 54)
Prolapse reduction during assessment	2	(46, 54)

^a^Some papers had more than one aim and were included in more than one group

##### Assessment methods for DO

Urodynamic assessment [50–55] trabeculation on cystoscopy [53] and artificial neural network analysis of clinical assessment [62] were used to assess for DO. However, even when other methods of assessment of DO were used, urodynamic assessment was carried out as the gold standard for comparison, despite no evidence that urodynamics are the gold standard.

##### The importance of urodynamic studies in the assessment of DO in patients with POP

Five studies were designed to evaluate the role of preoperative urodynamic assessment of DO (uninhibited detrusor contractions on a cystometrogram) in women with POP. Two studies examined the impact of urodynamic assessment on changing patient management [58, 59]. Two other studies examined the role of urodynamic assessment in predicting postoperative DO [50, 61] whereas the last study focused on the role of urodynamic assessment in diagnosing asymptomatic DO [51]. Not surprisingly, they came to different conclusions regarding the importance of preoperative urodynamic assessment in women with POP and two of the three found no benefit of urodynamic assessment in the preoperative evaluation of patients with POP.

##### Predicting post-repair overactive bladder

Three papers considered the preoperative risk factors for persistent or de novo overactive bladder (OAB; symptom of urinary frequency and urgency with or without the complaint of urgency incontinence) following surgical repair. Two studies used symptoms to assess for postoperative OAB [50, 57], one used urodynamic assessment post-operatively to assess for DO [60]. Pre-operative DO was not predictive of post-repair OAB or DO; however, one study found that preoperative OAB symptoms are more likely to resolve in the absence of preoperative DO [50].

##### Summary: assessment of voiding dysfunction in women with POP

Voiding dysfunction in patients with POP is common but evaluation techniques provide limited information.

1. The post-void residual volume estimation is commonly used for assessment of voiding dysfunction. The most commonly used value for diagnosing an elevated post-void residual is 100 ml by catheter or ultrasound.
2. Severity of POP is associated with reduced maximum and average flow rate, and voiding dysfunction is associated with the cystoscopic finding of trabeculation; however, there is no demonstrated benefit for using any of these methods in the routine assessment of the patient with POP.
3. Reduction of POP by pessary or packing often resolves voiding dysfunction and if this is noted on evaluation, it has a high predictive value for resolution of voiding difficulty after surgical POP repair.
4. Postoperative persistence of voiding dysfunction was found to be associated with diabetes, age, PVR >200 ml, P det max <10 and failure of a pessary to resolve voiding difficulty.
5. Preoperative urodynamic assessment was the most commonly utilized diagnostic tool for DO. Preoperative urodynamic diagnosis of DO did not change management, but the absence of preoperative urodynamic DO suggests that symptoms of OAB are more likely to resolve after surgery.

##### Further research

1. Further research is needed in the development of predictive models for persistence of voiding difficulty or DO postoperatively to aid in patient counseling.
2. Understanding how varying degrees of POP and how prolapse of different compartments affects voiding is poorly understood and needs further research.
3. Further study to assess the effect of voiding dysfunction on the patient both from a symptomatic and a morbidity perspective (recurrent UTIs, upper urinary tract disease) is not currently well understood

#### Assessment for SUI in women with POP

A substantial proportion of women presenting with POP report SUI symptoms. Preoperative SUI can either resolve or persist after POP surgery. Furthermore, a significant proportion of preoperatively continent women develop de novo SUI after POP surgery. SUI was addressed, either exclusively or as part of LUTS assessment, in 47 papers. Three main themes were identified: assessment of preoperative SUI, prediction of postoperative SUI, and prediction of de novo SUI.

##### Assessment of preoperative SUI

1. Stress test: the significance of patient position and prolapse reduction during the cough stress test was demonstrated in a study performed on 297 women waitlisted for POP surgery, with a third of them reporting SUI symptoms. Five different cough stress tests were performed with a subjectively full bladder (standing, semi-lithotomy, with and without reduction, reduction with speculum, and pessary). The test with the fewest positive results (34%) was the one performed without POP reduction in a semi-lithotomy position; the test with the most positive results (80%) was the one performed with pessary reduction in a semi-lithotomy position. With the full battery of tests, 93% of women with SUI symptoms demonstrated leakage; only 50% demonstrated leakage without reduction. Eighty-nine percent of the women with a positive stress test were diagnosed when performing at least two of the three tests with prolapse reduction, and 98% were diagnosed when performing all three tests with prolapse reduction (speculum and pessary reduction in the semi-lithotomy position, pessary reduction in the standing position). The authors also emphasized the importance of adequate bladder volume (200 ml) [63]. The findings were not compared with postoperative outcomes.
2. Q-tip angle: one study concluded that the Q-tip test is affected by POP. The angles were smaller with the prolapse reduced and with a full bladder [64]. A substantial correlation (r=0.68) between POP-Q point Aa and Q-tip angle was noted in a study on women presenting predominantly with SUI and anterior wall prolapse [65].
3. Importance of urodynamic studies in the assessment of preoperative SUI: one study concluded that a computer-based model including preoperative symptoms and patient’s baseline characteristics cannot predict preoperative urodynamic diagnosis and, as a consequence, cannot replace a preoperative urodynamic study [62]. In another retrospective study, preoperative urodynamic testing in patients with POP changed the management or counseling in only 3% (11 out of 316) in a cohort of women, with the indication for the study being OAB symptoms, mixed, or insensible urinary incontinence, or voiding difficulty (i.e., not occult SUI evaluation only). Major management alterations occurred mostly in women with SUI and concurrent voiding difficulty. The authors inferred that it might be in these patients that preoperative urodynamic study has its greatest value [58]. These two studies did not correlate the preoperative examination with postoperative outcomes.

##### Prediction of postoperative SUI

Postoperative SUI can represent persistent or de novo SUI. In this section, some studies approached postoperative SUI as persistent SUI [66] specifically, whereas some studies included women with any preoperative continence status and their results on postoperative SUI include both persistent and de novo SUI. De novo SUI specifically is addressed separately in the following section.

1. Predictive value of preoperative stress test: five studies provided data to calculate the predictive value of a negative stress test during preoperative urodynamic study for postoperative SUI in an unselected POP population (i.e., any preoperative continence status) [67–70]. All studies included stress tests with prolapse reduction. The negative predictive value ranged between 45 and 90% (median 78%; Table 7).

**Table 7:** Predictive value of a negative preoperative stress test for postoperative stress urinary incontinence after pelvic organ prolapse surgery

Reference	Type of surgery	Study design	Follow-up (months)	Baseline continence	*n*^a^	Preoperative test	Postoperative SUI outcome	Rate of postoperative SUI after a negative test, *n* (%)	NPV^b^ %
Alas et al. [67]	Any	Retrospective	Median 53	Any	274	UDS up to capacity with and without reduction (speculum)	Subjective (nonvalidated) or objective SUI (not specified)	27/274 (10)	90
Jeon et al. [68]	SCP	Prospective	24	Any	112	UDS up to capacity with reduction (swab)	Bothersome subjective SUI (UDI-6) or objective SUI (CST) or additional SUI surgery	32/112 (29)	71
Kasturi et al. [69]	TVM	Retrospective	6	Any	60	UDS with reduction (speculum or pessary)	Subjective (nonvalidated) and objective SUI (CST or UDS)	15/60 (25)	75
Leruth et al. [66]	SCP	Retrospective	Mean 25	Any	55	Stress test at capacity with and without reduction (manual) and UDS up to capacity with reduction (swab)	Subjective SUI (nonvalidated)	30/55 (55)	45
							Need for sling surgery	9/55 (16)	84
Park et al. [70]	SCP	Retrospective	Mean 11	Any	70	UDS up to capacity with reduction (pessary or speculum)	Need for SUI surgery	13/70 (19)	81

*SUI* stress urinary incontinence, *NPV* negative predictive value, *UDS* urodynamic study, *SCP* sacrocolpopexy, *CST* cough stress test, *TVM* transvaginal mesh, *UDI-6* Urinary Distress Inventory Short Form

^a^Only women without concomitant anti-incontinence surgery included

^b^Negative predictive value calculated based on numbers provided in the original studies

2. Other predictors for postoperative SUI:
  Three studies looked at other predictors of postoperative SUI. One study included only women with preoperative urodynamic SUI and the predictive urodynamic parameters for persisting urodynamic stress incontinence were overt (versus occult) urodynamic SUI, below normal maximum urethral closure pressure (MUCP, defined by the authors as <60 mmHg), and functional urethral length (FUL) < 2 cm [71].
  Two further studies included all women, regardless of preoperative incontinence status. The only two urodynamic parameters predictive of postoperative SUI in the one study were preoperative urodynamic stress incontinence and low P det Max [72]. In the other study, none of the investigated urodynamic parameters was associated with postoperative SUI [61].

##### Prediction model for postoperative SUI

A model developed to predict postoperative SUI for women regardless of preoperative continence status considers subjective urinary incontinence symptoms, stress test with and without prolapse reduction, age, point Ba, vaginal parity, and insertion of a mid-urethral sling during surgery [73]. The strongest predictor for postoperative SUI was preoperative SUI. The model’s ability to discriminate women at low or high risk for bothersome postoperative SUI or treatment for SUI during the first postoperative year was at a “useful level” (defined as area under the curve 0.76; interpretation: 0.5 not better than chance—1 perfect discrimination). However, the study does not report the extent to which the model correctly estimates the absolute risk (i.e., calibration), making it difficult to use it in counseling patients regarding operative options. Furthermore, our search did not identify any external validation studies for the model.

##### Prediction of postoperative SUI (occult SUI)

Occult SUI is defined as urine loss observed during a cough stress test with the POP reduced in a patient with POP who reports no urinary incontinence [74]. It is used as a preoperative test with the intention to identify women at risk of developing de novo SUI after prolapse surgery. Table 8 summarizes the studies that address the predictive value of occult SUI for de novo SUI.

**Table 8:** Diagnostic accuracy of occult stress urinary incontinence for de novo stress urinary incontinence after pelvic organ prolapse surgery without concomitant anti-incontinence surgery

Reference	Type of POP surgery	Study design	Follow-up (months)	Baseline continence	*n* ^a^	Test for OSUI	Definition of de novo SUI	Rate of de novo SUI		Sensitivity, %	Specificity, %	PPV, %	NPV, %
								Test positive, *n* (%)	Test negative, *n* (%)				
Alas et al. [67]	Any	Retrospective	Median 53	No subjective (nonvalidated) or objective SUI (stress test, UDS)	210	UDS up to capacity; reduction with speculum	Subjective (nonvalidated) or objective SUI (not specified)	N/A	12/210 (4)	N/A	N/A	N/A	94
Araki et al. [50]	TVM	Retrospective	6	No subjective SUI (ICIQ-UI + pad usage)	62	CST at 300 ml; reduction with gauze pack or pessary	SUI symptoms + pad usage	8/13 (62)	2/49 (4)	80	90	62	96
							Need for SUI surgery	3/13 (23)	0/49 (0)	100	83	23	100
Ballert et al. [75]	Any vaginal	Retrospective	Mean 17	No subjective (nonvalidated) or objective SUI (UDS)	24	UDS up to capacity; reduction with pessary or vaginal pack	Need for intervention for SUI	N/A	2/24 (8)	N/A	N/A	N/A	92
Costantini et al. [76]	SCP	RCT	6	No subjective (UDI) or objective SUI (stress test, UDS)	32	Stress test with full bladder; reduction both with fingers and speculum	Subjective (UDI) + objective SUI (stress test)	N/A	3/32 (9)	N/A	N/A	N/A	91
Ellström Engh et al. [77]	Vaginal NTR	Prospective	12	No subjective (nonvalidated) or objective SUI	74	CST at 300 ml; reduction with speculum	Subjective SUI (nonvalidated)	3/7 (43)	5/67 (7)	38	94	43	93
						Quantification test and 48-h pad test; reduction with pessary	Subjective SUI (nonvalidated)	2/6 (33)	6/68 (9)	25	94	33	91
Ennemoser et al. [78]	Any vaginal	Retrospective	Mean 68	No objective SUI (CST)	57	Stress test at 300 ml sitting and standing; reduction with speculum	Subjective (nonvalidated) and/or objective and/or treatment for SUI	16/57 (28)	N/A	N/A	N/A	28	N/A
							Need for SUI surgery	3/57 (5)	N/A	N/A	N/A	5	N/A
Goessens et al. [79]	Vaginal NTR	Retrospective	2	No bothersome subjective SUI (nonvalidated)	132	Subjective SUI (nonvalidated) revealed during ring pessary home test	Bothersome subjective SUI (nonvalidated) warranting treatment	N/A	12/132 (9)	N/A	N/A	N/A	91
Hafidh et al. [80]	Vaginal NTR	Retrospective	12	No subjective (nonvalidated) or objective SUI (UDS)	52	UDS; reduction with pessary/sponge stick	Subjective SUI warranting any treatment	N/A	2/52 (4)	N/A	N/A	N/A	96
Karateke et al. [81]	Vaginal NTR	Retrospective	20	No subjective SUI (UDI-6)	54	UDS up to 200 ml; reduction with two ring forceps bilaterally	Objective SUI (UDS)	N/A	8/54 (15)	N/A	N/A	N/A	85
Kleeman et al. [82]	Vaginal NTR	Retrospective	Mean 5	No subjective SUI (UDI)	53	CST, retrograde filling to subjectively full bladder; reduction with speculum	Subjective SUI (not specified)	N/A	1/53 (2)	N/A	N/A	N/A	98
Klutke and Ramos [83]	Vaginal NTR	Retrospective	Mean 42	No subjective SUI (nonvalidated)	20	UDS up to capacity; reduction with Gellhorn pessary	Subjective (nonvalidated) and objective SUI (UDS)	N/A	0/20 (0)	N/A	N/A	N/A	100
Liang et al. [84]	Vaginal NTR	Prospective	3–6	No subjective SUI (nonvalidated)	47	UDS up to capacity; reduction with pessary	Subjective SUI (nonvalidated)	11/17 (65)	0/30 (0)	100	83	65	100
Manodoro et al. [85]	Vaginal NTR	Retrospective	Mean 18	No subjective SUI (nonvalidated)	120	UDS up to capacity; reduction with ring pessary	Objective SUI (CST) or need for SUI surgery	10/43 (23)	15/77 (19)	40	65	23	81
							Need for SUI surgery	0/43 (0)	0/77 (0)	N/A	64	0	100
Misraï et al. [86]	SCP	Retrospective	Mean 20	No subjective (nonvalidated) or objective SUI (UDS)	53	UDS; reduction with sponge-holding forceps	Objective SUI (not specified) + pad usage	N/A	7/53 (13)	N/A	N/A	N/A	87
Reena et al. [87]	Vaginal NTR	Prospective	1.5	No subjective (nonvalidated) or objective SUI (not specified)	78	Pyridium pad test; reduction with ring pessary	Objective SUI (not specified)	34/53 (64)	0/25 (0)	100	57	64	100
Schierlitz et al. [88]	Vaginal NTR or SCP	RCT	6	No subjective SUI (nonvalidated)	39	UDS up to capacity; stress test with or without reduction; reduction with speculum or opened forceps	Need for SUI surgery	3/39 (8)	N/A	N/A	N/A	8	N/A
Sierra et al. [89]	Any including anterior/apical compartment	Retrospective	6	No objective SUI on clinical examination	97	UDS up to capacity; reduction with ring pessary, speculum, or scopette	Subjective SUI (nonvalidated)	N/A	2/97 (2)	N/A	N/A	N/A	98
Song et al. [90]	Vaginal NTR	Prospective	6	No subjective (nonvalidated) or objective SUI (CST)	206	Stress test with full bladder and 1-h pad test; reduction with ring pessary	Subjective SUI (UDI-6, IIQ-7)	18/45 (40)	30/161 (19)	38	83	40	81
							Need for SUI surgery	10/45 (22)	3/161 (2)	77	82	22	98
Srikrishna et al. [91]	Not specified	Prospective	24	No objective SUI (video-urodynamics)	48	Video-urodynamics up to capacity; reduction with ring pessary	Subjective SUI (KHQ) confirmed with video-urodynamics	2/5 (40)	1/43 (2)	67	93	40	98
Svenningsen et al. [92]	Any	Prospective	Mean 5	No subjective (not specified) or objective SUI (CST)	137	Manual reduction at 100ml	Subjective SUI (validated)	(40)	(16)	9	97	40	84
					135	Pessary reduction at 100 ml		(22)	(17)	9	94	22	83
					107	Pessary reduction at 300 ml		(50)	(13)	28	94	50	87
					79	Pessary 1 week		(47)	(11)	50	88	47	89
					74	Any of the above positive		(39)	(9)	73	71	39	91
Van der Ploeg et al. [93]	Any vaginal	RCT	12	Subjective SUI maximum once/week and no objective SUI (CST with full bladder)	182	Office evaluation stress test (subjectively full bladder) or urodynamics (up to maximum capacity); reduction with swab	Bothersome SUI (UDI), objective SUI (CST at 300 ml) and/or any treatment for SUI	18/47 (38)	11/135 (8)	62	81	38	92
Van der Ploeg et al. [94]	Any vaginal	RCT	12	Subjective UI maximum once/week and no bothersome UI (UDI)	172	Office evaluation stress test (subjectively full bladder); with or without reduction; reduction with swab on forceps	Bothersome SUI (UDI) and/or any treatment for SUI	9/32 (28)	7/140 (5)	56	85	28	95
					77	UDS up to capacity; with or without reduction; reduction with swab on forceps		3/22 (14)	4/55 (7)	43	73	14	93
Visco et al. [95]	SCP	RCT	3	Subjective SUI never or rarely (Medical, Epidemiological, and Social Aspects of Aging questionnaire)	48	UDS 300 ml; reduction with ring pessary with support	Subjective SUI (PFDI), objective SUI (stress test at 300 ml) and/or any treatment for SUI	1/2 (50)	19/46 (41)	5	96	50	59
					61	UDS 300 ml; manual reduction		4/8 (50)	18/53 (34)	18	90	50	66
					77	UDS 300 ml; reduction with swab		11/14 (79)	22/63 (35)	33	93	79	65
					49	UDS 300 ml; reduction with forceps		4/8 (50)	20/41 (49)	17	84	50	51
					62	UDS 300 ml; reduction with speculum		11/20 (55)	17/42 (40)	39	74	55	60
					297^b^	All methods		31/52 (60)	96/245 (39)	24	88	60	61
Wei et al. [96]	Any vaginal	RCT	3	No subjective SUI (PFDI)	170	Stress test at 300 ml; reduction with swab	Stress, urgency, or mixed UI defined as a positive CST, bothersome symptoms, and/or treatment for UI	41/57 (72)	43/113 (38)	49	81	72	62
Yamada et al. [97]	Anterior colporrhaphy	Retrospective	Mean 58	No subjective (nonvalidated) or objective SUI	10	1-h pad test and stress test; reduction with ring pessary or vaginal pack	Subjective SUI (nonvalidated)	N/A	0/10 (0)	N/A	N/A	N/A	100

Diagnostic accuracy (sensitivity, specificity, positive predictive value, negative predictive value) of occult stress urinary incontinence for de novo stress urinary incontinence is shown. Some of the original studies reported diagnostic accuracy values; some studies provided rates of de novo SUI after positive and/or negative test, and diagnostic accuracy values were calculated based on these data

*POP* pelvic organ prolapse, *OSUI* occult stress urinary incontinence, *SUI* stress urinary incontinence, *PPV* positive predictive value, *NPV* negative predictive value, *UDS* urodynamic study, *N/A* not applicable, *TVM* transvaginal mesh, *CST* cough stress test, *SCP* sacrocolpopexy, *RCT* randomized controlled trial, *NTR* native tissue repair, *UI* urinary incontinence, *ICIQ-UI* International Consultation on Incontinence Questionnaire–Urinary Incontinence, *UDI* Urinary Distress Inventory, *UDI-6* Urinary Distress Inventory Short Form, *IIQ-7* Incontinence Impact Questionnaire Short Form, *KHQ* King's Health Questionnaire

^a^Only women without concomitant anti-incontinence surgery were included

^b^Total number of women 165; each subject underwent two different prolapse reduction methods

Twenty-five studies provided either the diagnostic accuracy measures or data enabling the calculation for positive and/or negative test [50, 67, 75–97]. Baseline continence status, diagnostic criteria for occult SUI, methods to reduce the prolapse, surgical procedures, and the definition of de novo SUI varied widely among the studies, making the comparison challenging. Most studies defined occult SUI clearly as SUI demonstrated only during prolapse reduction, whereas some also included demonstrable urodynamic SUI without prolapse reduction in symptomatically continent women. The diagnostic accuracy of occult SUI differed greatly, likely because of the heterogeneity in the studies. The medians (and ranges) for sensitivity were 39% (5–100), for specificity they were 86% (57–97), for positive predictive value they were 40% (0–79), and for negative predictive value they were 91% (51–100).

##### Importance of urodynamic studies for diagnosis of occult SUI

One study reported similar occult SUI rates with stress testing during physical examination and urodynamic studies. In 76%, occult SUI was identified with both tests, in 11% with urodynamic studies only, and in 13% during physical examination only (kappa 0.648). They did not correlate the findings with postoperative de novo SUI rates [98].

Another study compared the predictive value of demonstrable SUI during basic office evaluation versus urodynamic study for de novo SUI. Stress tests were performed in the lithotomy position with (swab on forceps) and without reduction of the prolapse. During basic office evaluation women were examined with a subjectively full bladder and during urodynamic studies with 300-ml bladder filling and at maximal bladder capacity. More women demonstrated SUI during urodynamic study, but the diagnostic accuracy for bothersome de novo SUI or treatment for de novo SUI was not improved by the addition of the urodynamic study [94].

##### Other predictors of de novo SUI

Two studies were aimed at identifying other risk factors for de novo SUI. Urodynamic markers that were associated with de novo SUI were low MUCP [99], low FUL [99], and bladder outlet obstruction [100].

Two studies demonstrated that occult SUI is also seen in posterior wall prolapse [101, 102] up to the same extent as with anterior wall prolapse [101].

##### Prediction model for de novo SUI

A model and risk calculator developed to predict de novo SUI among women without preoperative SUI symptoms contains seven predictors: age, number of previous vaginal births, urine leakage associated with urgency, history of diabetes, BMI, preoperative reduction stress test result, and placement of a midurethral sling during surgery. The model predicted absolute risk accurately, with slight tendencies to overestimate the risk when the probability reached 50% or greater. The concordance index (interpretation: 0.5, not better than chance to 1, perfect discrimination) was 0.73 in the original study [103], and it outperformed both expert opinion and preoperative stress testing in discriminating between women who developed de novo SUI during 12 months follow-up and not. However, when the model was applied to other samples (external validation), the results for the concordance index or area under the curve decreased to 0.62, 0.63, and 0.69 [103–105]. One study assessed the model’s performance as a diagnostic test using a probability of de novo SUI of >50% as a cut-off for a positive test. Using this cut-off, a positive test had a predictive value of 27% (i.e., 27% of women with an estimated risk of 0.5 or higher actually developed SUI). This illustrates how the model overestimates the risk when the baseline risk is lower than in the original sample [105].

##### Summary: assessment of SUI in patients with POP

The evaluation of SUI in patients with POP is very complex and recommendations vary widely.

1. In women with POP and SUI, the cough stress test should be performed with at least 200-ml bladder volume and with the prolapse reduced either with a speculum or pessary in order to have the highest chance of identifying a positive result.
2. Assessment of UDS in women prior to POP surgery has been shown to change management in a small percentage of cases, for example, when SUI (clinical or occult) coexists with voiding dysfunction. The management may change by the avoidance of a concomitant continence procedure or the choice of one with a perceived lower risk of associated voiding dysfunction.
3. There are no comparative data on different diagnostic alternatives correlating with postoperative outcomes as studies such as VALUE [106] and VUSIS [107] excluded women with prolapse beyond the hymen.
4. In an unselected POP population, a negative reduction stress test during preoperative urodynamic assessment has a median negative predictive value of 78% (range 45–90%) for postoperative SUI. There is conflicting evidence regarding the predictive value of further urodynamic parameters such as MUCP and FUL.
5. More preoperatively continent women will demonstrate occult SUI during a urodynamic assessment compared with office evaluation stress test but this does not have greater accuracy for bothersome de novo SUI or treatment for de novo SUI. The demonstration of preoperative occult SUI during urodynamic assessment has a positive predictive value for de novo SUI of 40% (0–79%) and its absence has a negative predictive value of 91% (51–100%) respectively.
6. A de novo SUI prediction model that incorporates seven variables and outperforms pure chance, expert opinion, and reduction cough stress test alone. However, in follow-up studies the model performed poorly, overestimating the risk when compared with the original study.

To sum up, the most useful information from the evaluation of a patient with POP with regard to postoperative stress incontinence is the high negative predictive value of a negative stress reduction test.

##### Further research

1. Future research should look to improve the performance of current prediction testing, and develop new predictive parameters. These could probably be identified by deepening our understanding of the biological and biomechanical explanations behind de novo and persistent SUI.
2. The prognostic value of MUCP and FUL should be re-assessed in further studies.
3. Persistent and de novo SUI probably have different prognostic factors, thus developing separate models may be feasible and increase accuracy.
4. Researchers should follow ﻿The Transparent Reporting of a Multivariable Prediction Model for Individual Prognosis or Diagnosis statement when presenting a new or validating an existing prediction model [108].

#### Evaluation of hydronephrosis and hematuria in patients with POP

There were two studies that discussed the prevalence of hydronephrosis and hematuria in women with POP. The study on hydronephrosis evaluated 180 patients and found some degree of hydronephrosis in 30%. A multivariate statistical analysis revealed only the two following factors associated with hydronephrosis. First, anterior compartment prolapse, as defined by POP-Q point Ba; noting that for every 1-cm increase, the relative risk of hydronephrosis increases by 1.68. Second, cystometric capacity; it was found that every 100-ml increase in maximum cystometric capacity increases the relative risk of hydronephrosis by 1.5. However, the model only predicted about 30% of the hydronephrosis [109].

The study evaluating microscopic hematuria (defined as ≥ red blood cells per high power field) noted its presence in 20.1% in a population of 1,040 women. This population is at a very low risk of urinary tract malignancy and the authors suggested that the cut-off for significant microscopic hematuria in this population should be re-evaluated [110].

To summarize: the severity of anterior vaginal wall prolapse and cystometric capacity are associated with hydronephrosis in a limited number of studies; prediction models are not well developed.

### Assessment of gastrointestinal tract symptoms in women with POP

**Fig. 3:**
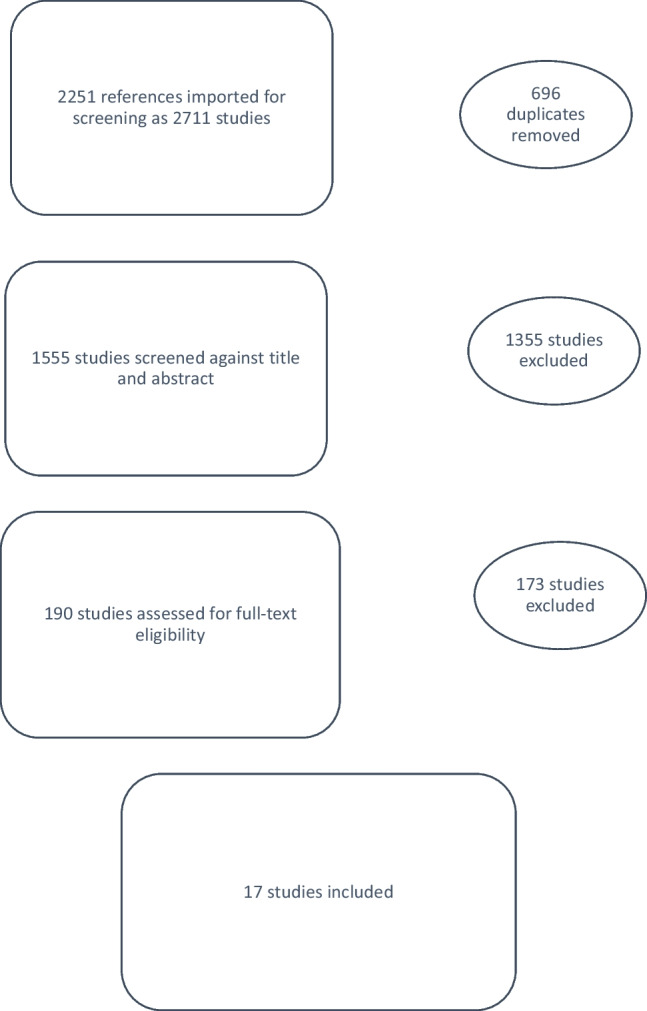
Preferred Reporting Items for Systematic Reviews and Meta-Analyses diagram for gastrointestinal radiographic/physiological testing

A review of the existing literature on the assessment of GIT symptoms in women with POP identified 2,251 titles and abstracts, of which 17 studies were included in the final review of this section (Fig. 3). Studies were included whose primary population or a significant portion of the study population were women with POP, who then underwent evaluation of the GIT other than or in addition to symptom assessment and clinical examination (Table 9).

**Table 9:** Evidence table for the evaluation of prolapse in women with symptoms of obstructed defecation and anal incontinence

Reference	Study design	Population	Method(s) of clinical assessment	Results						Discussion
Fluoroscopic defecography										
Kelvin et al. [111]	Retrospective cohort study, USA	*n*=170 consecutive women with symptoms of pelvic floor dysfunction referred for dynamic cystoproctography	BW ±POP-Q	Only 74% POP-Q—POP-Q not used for analysis						Descriptive study showing that cystoceles and rectoceles are similarly diagnosed by both BW and DCP, but more enteroceles diagnosed by examination
				Findings on BW vs DCP						
						BW	DCP	Both		
				Rectocele		91%	76%	70%		
				Enterocele		40%	28%	14%		
			DCP	Cystocele		81%	94%	78%		
Kaufman et al. [112]	Retrospective descriptive cohort study, USA	*n*=22 women with symptomatic prolapse went on to pelvic reconstructive surgery	Questionnaires	86% had previous pelvic surgery						Low concordance of findings between modalities
				41% patients had a change in surgical plan owing to imaging results						
				POP-Q vs DCCP vs dMRI						
						POP-Q	DCCP	dMRI		
				Cystocele		68%	45%	41%		
				Rectocele		86%	82%	50%		
			Physical examination, POP-Q	Enterocele		36%	41%	36%		
			DCCP	Sigmoidocele		0%	9%	0%		
			Had defecography phase	Levator ani defect		0%	0%	18%		Levator ani defects only diagnosed by dMRI
			dMRI	Internal rectal prolapse		9%	45%	0%		Sigmoidocele only diagnosed by DCCP
			No defecography phase	Full-thickness rectal prolapse		9%	0%	0%		Lack of defecography phase during MRI likely contributes to findings
Lopez et al. [113]	Prospective cohort study, Sweden	*n*=25 women with POP on clinical examination planning to undergo surgery	Clinical examination (no POP-Q or BW)	No statistical analysis						Descriptive study suggesting CDP might contribute to characterization of prolapse, but limited by lack of use of either POP-Q or BW and small numbers
				Pre-operatively: cystocele on clinical examination vs CDP 28% vs 88%						
			Questionnaires	Pre-operatively: rectocele on clinical examination vs CDP 96% vs 84%						
			CDP	Enterocele on clinical examination vs CDP 8% vs 24%						CDP may be helpful in diagnosing enteroceles
Takano and Hamada [114]	Prospective cohort, Japan	*n*=66, which included 55 female patients, 11 male patients	Clinical examination (no POP-Q or BW)	No statistical analysis						Descriptive study limited by lack of statistical analysis, lack of POP-Q or BW, mixed sex population, and lack of defecography phase
				75% of patients with symptoms of vaginal prolapse showed descent of the vagina on DCR						
			DCR: opacification of the ileum, bladder, vagina, rectum, and perineum	78% of patients with uterine descent had an enlarged angle between the vaginal axis and horizontal line at the superior border of the sacrum on DCR						
			No defecography phase	*68% of patients with symptoms of descent of the rectum or obstructed defecation had descent of the rectum on DCR						
		Symptoms of prolapse, defecatory dysfunction, incontinence NOS		*Female + male						Study does not demonstrate benefit of DCR
Roovers et al. [115]	Prospective cohort study, The Netherlands	*n*=82 women with symptomatic stage II or greater prolapse, planned for surgery	Physical examination, POP-Q	Abnormal defecography was defined as presence of an enterocele, rectal intussusception, or both						Applying the proposed scoring system may predict the probability that an enterocele or rectal intussusception is found; for scores >1 and <8, defecography may be more useful i.e., for patients with some predictive factors but not others, imaging may add to clinical assessment
				Abnormal= 32%						
				Enterocele = 28%						
				Rectal intussusception 11%						
				Both = 7%						
				History of pelvic surgery, size of the posterior vaginal wall prolapse, and the presence of constipation predicted abnormal defecography (i.e., enterocele, rectal intussusception)						
				Used to create a formula to predict probability = 3+3 × history of pelvic surgery* + Ap (in cm) +3 × constipation*						
			Questionnaires (standardized)	*Present = 1, absent = 0						
			Fluoroscopic defecography with vaginal also opacified	Patients with probability less than 20% (score ≤1) or greater than 70% (score ≥8) of abnormal defecation do not get additional information from this study						
Groenendijk et al. [116]	Prospective cohort study, The Netherlands	*n*=59, women with primary pelvic organ prolapse: 68 enrolled; 5 dropped out; 4 defecography incorrectly performed	Physical examination, POP-Q	No significant relationship was found between defecatory symptoms and presence of posterior vaginal wall prolapse on examination (*p*=0.33), rectocele (*n*=0.19), or enterocele (*n*=0.99) on defecography						Clinical examination may overestimate posterior vaginal wall prolapse and underestimate enterocele
			DDI							
			Fluoroscopic defecography with vagina also opacified	Clinical examination diagnosis of a rectocele compared with defecography						
				Sensitivity 1.0, 95% CI 0.82 to 1						
				Specificity 0.23 95% CI −0.11 to 0.38						
				Clinical examination diagnosis of enterocele compared with defecography						
				Sensitivity 0.07, 95% CI 0.002 to 0.32						
				Specificity 0.95, 95% CI 0.85 to 0.99						No correlation of bowel symptoms with posterior wall prolapse on examination or rectocele or enterocele on defecography
Kim et al. [117]	Prospective cohort, South Korea	*n*=109	Physical examination, POP-Q	Physical examination did not diagnose enterocele, sigmoidocele, or RI						Clinical examination misses enteroceles, sigmoidoceles, and rectal intussusception found by DCCP
				The surgical plan changed in 22% of cases						
				Patients with a changed surgical plan had a higher prevalence of bowel symptoms (*p*=0.023						
				Findings on examination vs DCCP						
					Examination	DCCP		Total	*p* value	
						Negative	Positive			
				Cystocele total	Negative	20	10	30	<0.001	
						0	79	79		
					Positive	20	89	109		
		113 enrolled		Rectocele total	Negative	27	32	59	<0.001	
						0		50		
					Positive	27	5,820	109		
		4 dropped out		RI total	Negative	101	8	109		
						0	0	0		
					Positive	101	8	109		
		Women with stage ≥II POP and urodynamic confirmed urinary incontinence	DCCP	Enterocele total	Negative	107	2	109		
						0	0	0		
					Positive	107	2	109		
		Without HO pelvic reconstructive surgery	Bladder and vaginal = also opacified	Sigmoidocele total	Negative	105	4	109		This may influence surgical planning
						0	0	0		
					Positive	105	4	109		
Vanbeckevoort et al. [118]	Prospective cohort study, Belgium	*n*=35 women with clinical evidence of pelvic floor descent	Clinical examination (no POP-Q or BW)	All patients underwent both imaging studies						Cystocele, vaginal vault prolapse, rectocele, enterocele, and rectal descent most readily seen on CCD with voiding and defecation phase
				Compared with MRI, CCD II diagnoses additional defects:						
				Cystocele = 14						
				Vaginal vault prolapse = 20						
				Enterocele = 4						
			CCD with (CCDII) and without (CCDI) a voiding and defecation phase	Rectocele = 13						
			Dynamic, single-shot MRI sequence without defecography phase	Rectal descent = 5						
MR defecography										
Hausammann et al. [ 119]	Prospective cohort study, Switzerland	*n*=37 women	BW	2/3 patients had moderate to large rectocele on MRD						Patients with a rectocele on examination may have other pelvic floor defects as well
				No significant association between size of rectocele on MRD and constipation or fecal incontinence						
				67.5% of women with a rectocele had a concomitant intussusception						
				Significantly more likely to have an enterocele (*p*=0.013)						
			Symptom questionnaires (Cleveland clinic constipation score and Wexner faecal incontinence score)	Obstructed defecation symptoms did not differ between isolated rectocele and rectocele + intussusception						
		Patients with rectocele and defecatory dysfunction	MRD (open)	Higher grade intussusception was associated with FI (*p*=0.048)						
Aziz et al. [120]	Case series	*n*=7 patients with pelvic floor disorder symptoms and a history of cystectomy and hysterectomy referred for MRD	Physical examination	5 POP-Q stage II or III						MRD may be useful in post-cystectomy patients with vaginal bulge
				2 POP-Q stage 0						
				MRD findings:						
			POP-Q	4 patients = anterior enterocele (small bowel), moderate						
			MRD	3 patients = anterior sigmoidocele, moderate						Study limited by very specific population
Pollock et al. [121]	Retrospective cohort study,	*n*=54 women	Physical examination	Symptoms						MRD not significantly correlated with BW
				96% bothersome POP						
				Spearman correlation coefficient between MRD grading compared with examination						
						Overall		Anterior wall		
		170 patients with POP screened	POP-Q or BW	BW		rho −0.001; *p*=0.998		rho 0.197; *p*=0.154		Overall POP-Q stage and anterior wall correlated positively and significantly with MRD
		116 excluded because of incomplete examination or MRD information	MRD	POP-Q		rho 0.305; *p*=0.025		rho 0.436; *p*=0.001		MRD may provide different information than clinical examination, particularly BW staging
Lin et al. [122] (same group as Pollock et al. [121])	Retrospective cohort study, USA	*n*=178	Physical examination, BW	Patients with POP specifically not reported						MRD may provide additional information on the presence of an enterocele
				Physical examination compared with MRD for enterocele detection						
				Sensitivity 0.300, specificity 0.926						Anterior wall had the best correlation between examination and MRD
				Spearman correlation coefficient between MRD grading and BW grading				Agreement between BW grade 3,4 and MRD moderate to severe		Findings impacted by how MRD grading is defined
				Anterior		rho=0.652, moderate positive		84.6%		
		274 patients with POP or other pelvic floor disorder underwent MRD		Apical		rho=0.195, poor		63%		
		96 excluded for male sex, incomplete examination or MRD, inability to defecate rectal gel	MRD	Posterior		rho=0.277, poor		78.7%		
Arif-Tiwari et al. [123] (same group as Lin et al. [122] and Pollock et al. above [121])	Retrospective cohort study, USA	*n*=237	Dynamic MR with Valsalva only vs defecography phase	56% prior surgery for POP or UI Vaginal prolapse 22.8%						Suggests that dynamic MRI for patients with POP should include defecography phase
				67.4% prior hysterectomy						
				0% prolapse detected by Valsalva only but not defecography phase						
				Percentage POP detected by defecography phase but not Valsalva only:						
				Cystocele 37.6%						
				Rectocele 25.7%						
		274 with symptoms of POP		*p*<0.0001						
		37 patients excluded for male sex or inability to tolerate or defecate rectal gel	No physical examination data							
Faucheron et al. [124]	Prospective cohort study, France	*n*=50 patients with posterior vaginal wall prolapse who ultimately had surgical repair	Physical examination	Peritoneocele						MRD and DCCP had good interobserver agreement for rectocele and posterior colpocele
				DCCP						
				Sensitivity 0.833; specificity 1.000						
			POP-Q	MRD						
			DCCP	Sensitivity 0.633, specificity 1.000						
			MRD	Detection of defects and interobserver agreement of findings at surgery and radiographic findings						
			Findings at surgery		DCCP		MRD			
			Studies undergone by all patients but not reported on:	Posterior colpocele	89%; kappa=0.69, good		91%; kappa=0.76, good			
			Colonic transit time study	Rectocele	91%; kappa=0.69, good		93%; kappa=0.79, good			
			Anal manometry	Peritoneocele	87%; kappa=0.72, good		76%; kappa=0.54, moderate			
			Endoanal US	Full-thickness rectal prolapse	95%; kappa=0.80, good		91%; kappa=0.56, moderate			
			Colonoscopy	Internal rectal prolapse	93%; kappa=0.85, excellent		87%; kappa=0.69, good			DCCP was better at detecting peritoneocele, full-thickness, and internal rectal prolapse, possibly because of more physiological positioning for DCP
Lienemann et al. [125]	Case–control study, Germany	*n*=66	Physical examination	Diagnosis of enterocele	Examination		MR-CCRG			MR-CCRG was better than DCP at diagnosing enteroceles
				Present	43		53			
				Absent	12		2			
				Diagnosis of enterocele	Examination		DCP			
				Present	23		14			
				Absent	11		20			
				Diagnosis of enterocele	MR-CCRG		DCP			
		55 patients with POP	DCP	Present	29		14			
		11 controls without POP	MR-CCRG	Absent	5		20			MR-CCRG detected enteroceles missed on examination
Anal physiology testing										
Groenendijk et al. [126]	Prospective cohort study, The Netherlands	*n*=59 women with primary POP stage ≥II	Symptom questionnaire (defecation distress inventory)	Patients with POP vs health controls reference values						AFT and AES add limited information to the routine evaluation of POP patients.
				Lower squeezing pressure						
				Delayed first sensation, desire, capacity						
				Prolonged PNTLT						
				*p*<0.01						
		68 enrolled	POP-Q	Patients with FI had significantly lower resting (*p*=0.036) and squeezing pressures (*p*=0.046) and increased risk of external sphincter defect						
		4 dropped out	AFT: manometry, sensation, PNTLT	OR= 12.75; 95% CI 2.40–66.67						
		5 had testing done incorrectly	AES	Manometry was not different between patients with and without constipation						
				Anorectal sensation and sensitivity were not related to the stage of posterior wall prolapse						Patients with fecal incontinence may benefit from this testing
Zbar et al. [127]	Prospective cohort study, UK	*n*=73 women (14 isolated rectocele aka type 1, 26 rectocele and apical POP aka type 2, 33 controls)	BW	All patients with rectocele had this finding on examination and defecography						There are few consistent, differences in anal physiology between isolated rectoceles and those associated with other prolapse
				Reduced resting and squeeze pressure in type 2 rectoceles						
				*p*<0.001						
				Elevated resting pressure in type 1 rectocele						
				*p*<0.001						
				But squeeze pressure not significantly different						
				Reduced inhibitory slope in RAIR measurements in both rectocele types compared with controls (type 1 *p*<0.001, type 2 *p*=0.002)						
			Defecography	Maximum inhibitory pressure lower in type 1						
			Anorectal manometry, vector manometry, parametric assessment of the rectoanal inhibitory reflex	*n*=0.006						

*BW* Baden–Walker prolapse grading system, *POP-Q* Pelvic Organ Prolapse Quantification, *DCP* dynamic cystoproctography, *DCCP* dynamic cystocolpoproctography, *dMRI* dynamic magnetic resonance imaging, *CDP* cystodefecoperitoneography, *NOS* not otherwise specified, *DCR* dynamic contrast roentgenography, *DDI* defecation distress inventory, *HO* heterotopic ossification, *CCD* colpocystodefecography, *MRD* magnetic resonance defecography, *FI* fetal incontinence, *MR-CCRG* magnetic resonance colpocystorectography, *PNTLT* pudendal nerve terminal latency time, *AFT* anorectal function testing, *AES* anal endosonography, *RAIR* renoanal inhibitory index

#### Defecography

Several studies compared various defecography imaging modalities with each other [112, 118, 124, 125]. Difficulties in evaluation of the existing literature included the use of various methods for the assessment of prolapse on physical examination, including the Baden–Walker halfway system, the POP-Q system, and several manuscript-specific nonstandardized examination techniques. In addition, various methods of performing the imaging and interpretation of results were described. In studies of fluoroscopic defecography, there was variability in which compartments were opacified with contrast; although the rectum was universally opacified, other possible compartments included the bladder, vagina, perineum, peritoneum, and small bowel.

Three studies of fluoroscopic defecography found that this imaging modality detected more enteroceles than physical examination [111, 113, 117]. Two studies found that MRI defecography was able to diagnose enteroceles more readily than physical examination, and one of these found that MRI defecography was also able to diagnose more enteroceles than fluoroscopic defecography [122, 125]. Two studies found that sigmoidoceles were not diagnosed on examination but were identified by fluoroscopic defecography [112, 117]. One study found that the size of the posterior vaginal wall prolapse, as assessed by physical examination, was associated with the finding of enterocele and/or rectal intussusception on fluoroscopic defecography [114].

Patient symptoms were assessed in two studies that found that defecatory symptoms were not significantly associated with findings on radiographic imaging or examination [115, 116]. One study found no relationship between defecatory symptoms in women with posterior vaginal wall prolapse and abnormal defecography. The other found no relationship between defecatory symptoms and posterior vaginal wall prolapse on examination or rectocele or enterocele on defecography [115, 116]. One study found that two thirds of women with a rectocele and symptoms of obstructed defecation or anal incontinence had intussusception (13.5% Oxford Grade I, 41% Grade II, and 13.5% Grade III) on MR defecography and were more likely to have an enterocele [119].

#### Anal physiological testing and anal ultrasound versus physical examination

Anal physiology and anorectal endosonography testing added limited information to the routine physical examination evaluation of POP patients for identifying intussusception [126, 127].

Patients with fecal incontinence may benefit from this testing. In terms of the clinical consequences of the imaging investigation, two studies found that the imaging results led to a change in surgical plan for 22–41% of patients [112, 117].

#### Definitions/interpretation of radiographic imaging studies

Consensus on definitions and interpretations of fluoroscopic defecography and MRI defecography have been developed by multiple stakeholder societies including the IUGA [128, 129]. Although these documents represent consensus on the use of these imaging modalities in patients with defecatory disorders, they “do not” contain information pertinent to patients with pelvic organ prolapse regarding specific methods and measurements. There is no consensus on whether or not patients with prolapse and no GI symptoms should undergo any testing beyond a thorough physical examination. It has been agreed upon that imaging should include measurements performed during the defecation phase rather than only with strain to improve sensitivity [123, 128, 129]. Studies in which there was no defecography phase have limited applicability.

##### Summary: assessment of GIT symptoms in women with POP

Summary of supplemental evaluation for GI dysfunction in women with POP is an area requiring a significant amount of research before any concrete recommendation can be made.

1. There were no studies that reported on patient outcomes in those evaluated by fluoroscopic defecography, MRI defecography, or anal physiology testing, and those who did not undergo this evaluation. Therefore, the clinical significance of this testing, particularly in asymptomatic patients, remains uncertain. It does seem that some anatomical defects, including enterocele, sigmoidocele, and intussusception, are better visualized with either fluoroscopic defecography or MRI defecography, but how this relates to clinical decision-making or more specifically outcomes, remains unclear.
2. In patients where these diagnoses are in question or in patients who present with GI symptoms, it is reasonable to obtain further imaging and testing beyond a routine clinical examination. However, these additional studies can be expensive and uncomfortable to patients, and currently there is no apparent benefit to identifying an underlying condition that would influence treatment decisions and outcomes. Until a benefit is established, their routine use in asymptomatic women with POP should be discouraged outside of research protocols.

##### Further research

Future studies comparing imaging and physiological testing with clinical examination need to compare their results with standardized clinical evaluation in the form of the POP-Q. Standardized minimum criteria for imaging and physiological testing need to be established, as well as a standardized reporting system to allow for comparison between studies. Until these are drawn up it will remain almost impossible to evaluate the literature.

Studies in patients with POP and no GI complaints comparing radiographic/physiological testing with no testing need to be evaluated with meaningful outcome measures.
